# Continuing Medical Education Delivery Preferences Among Physicians and Advanced Practice Providers in Emergency Medicine

**DOI:** 10.7759/cureus.20406

**Published:** 2021-12-14

**Authors:** Andrew Kalnow, Jennifer Beck-Esmay, Jeffrey Riddell, John Casey, Jestin N Carlson, Salim R Rezaie, Andrew Little

**Affiliations:** 1 Emergency Medicine, OhioHealth Doctors Hospital, Columbus, USA; 2 Emergency Medicine, Ohio University Heritage College of Osteopathic Medicine, Athens, USA; 3 Emergency Medicine, Mount Sinai St. Luke's - Mount Sinai West, New York, USA; 4 Emergency Medicine, University of Southern California Keck School of Medicine, Los Angeles, USA; 5 Emergency Department, Saint Vincent Hospital, Erie, USA; 6 Emergency Medicine, Methodist Hospital, San Antonio, USA; 7 Emergency Medicine, AdventHealth East Orlando, Orlando, USA

**Keywords:** provider education and collaboration, emergency medicine, education delivery, continuing medical education, medical education

## Abstract

Study objective

We investigate the continuing medical education delivery preferences among emergency medicine providers, both physicians and advanced practice providers (APPs), within a large, national emergency medicine group.

Methods

A survey was sent via email to all emergency medicine health care providers in the practice group, including questions evaluating both delivery method and topic preference for continuing education. The study was sent to providers whom the group employed from October 2019 through January 2020.

Results

A total of 2038 providers, 1373 physicians, and 665 APPs completed the survey - a completion rate of 68.7%. In general, practitioners expressed willingness to learn across various platforms, with a strong overall preference towards online and on-demand options, including video, podcast, and written materials. Universally, a larger percentage of APPs identified a desire for more continuing education across all topics than physicians.

Conclusion

Education preferences among emergency medicine providers vary with a strong trend toward online and on-demand content. Understanding the delivery and topic preferences for providers is important for the optimal creation of continuing education content.

## Introduction

Background

Continuing medical education (CME) delivery has become increasingly complex, with multiple platforms available for content distribution. Understanding how practicing health care providers prefer to receive education, including CME, is critical to offering high-quality content in the format most desired by the end user. Over the past two decades, the delivery of education has transitioned from primarily live and print content to include on-demand options such as podcasts, videos, vlogs, and blogs [1]. During this same period, CME delivery became a competitive space for content over these emerging platforms. This trend in CME offerings has paralleled the general growth of alternative “online” media delivery platforms. For example, from 2006 to 2020, the percentage of the United States population that has listened to at least one podcast increased from 11% to 55%, a 400% increase [2]. Younger physicians favor CME content via virtual online options while older physicians prefer CME via live, in-person meetings [3].

Importance

Online education delivery models appear attractive to emergency physicians, whose inconsistent schedules and shift lengths limit their ability to engage in structured education [4]. These clinicians face unique challenges in fulfilling CME requirements for states, specialty societies, certification, and subspecialty certification. Many providers entering early practice demonstrate a strong preference for on-demand learning via asynchronous learning mechanisms. Podcasts, blogs, and vlogs (ie, short, high-yield videos explaining various topics) provide unique education opportunities without the requirement for time or location-based access [5-6]. Many physicians spend up to four hours per week engaged in podcast educational material, and a majority have endorsed them as being more beneficial to learning than textbooks or journal articles [5]. Furthermore, physicians indicate that education received via podcasts and blogs may potentially change their current clinical practice. Data characterizing the education preferences of advanced practice providers (APPs) are lacking [2,6]. Currently, no data convey how emergency physicians and APPs would like to be taught during CME activities. 

Goals of this investigation

Understanding current trends in CME preferences among practicing physicians and APPs could offer key information to guide the growth and expansion of content and provide insight into best practices for revision and improvement. This will allow content creators to tailor future educational offerings, allowing for high-quality content to be created and delivered in the preferred modality. Whereas previous studies addressed education use among resident learners across various specialties, there is little published research on CME preferences among graduate emergency medicine practitioners. Furthermore, there is a gap in the literature regarding CME preferences of advanced practice providers or the difference in preferences between different types of clinicians within the same specialty. We conducted this study to evaluate the continuing educational preferences of practicing physicians and APPs in emergency medicine.

## Materials and methods

Study design, setting, and participants

We performed a survey-based study to evaluate the educational needs and desires of a cohort of physicians and APPs from around the United States whom a national emergency medicine group employed from October 2019 through January 2020. This group employs clinicians who practice emergency medicine, pediatric emergency medicine, and other specialties. Those who practiced emergency medicine represented a group of clinicians who work in various clinical settings, including community, tertiary, and critical access, with fewer than 10% working in academics.

Outcomes and survey development

The survey was developed by a group of educators (including author JC) to discover the above-mentioned providers’ educational needs and preferences. The survey sent to providers included 11 questions, seven of which were specific about an existing learning management system. These items were not included in our analysis. The remaining four questions were designed to elicit responses about providers’ preferences of CME in terms of topics to be covered and specific delivery models. The complete survey is available in Appendix 1.

An email containing the survey was sent to all physicians and APPs employed by the practice group. Weekly email reminders to complete the survey were also sent for 10 weeks. Participants were only allowed to complete the survey once. Answers were anonymous, and the survey was required to be completed in one attempt. There were no incentives given for the completion of the survey. The OhioHealth (Columbus, OH) institutional review board approved this study before performing any statistical analysis.

Analysis

Fisher’s tests were used to compare groups by preferred topics and preferred method of receiving education. A retrospective sample size calculation was performed, and the data set was found to be powered correctly for analysis. For this study, statistical significance was set at p < 0.05.

## Results

The survey was sent to 2967 emergency medicine providers, and 2038 (68.7%) completed the survey. Of these, 1373 were physicians (67.37%) and 665 were APPs (32.6%). This response distribution matches the full-time distribution of providers in the group, 66% physician and 33% APP.

Regarding preferred methods of receiving educational material, both groups preferred video and podcasts over classroom teaching and webinars (Table 1). There were several significant differences between the preferences of physicians and APPs. APPs preferred videos, classroom instructor-led training, and live webinars significantly more than physicians.

**Table 1:** Preferred methods of receiving educational material among community physicians and APPs APP: advanced practice provider

Educational Material	Total (n=2038)	Physician (n=1373)	APP (n=665)	p-value
Videos	1383 (67.86%)	901 (65.62%)	482 (72.48%)	0.002
Podcasts	961 (47.15%)	655 (47.71%)	306 (46.02%)	0.478
Presentation Slides With Audio Narration	862 (42.3%)	586 (42.68%)	276 (41.5%)	0.633
Written Material/Articles	722 (35.43%)	504 (36.71%)	218 (32.78%)	0.084
Infographics	284 (13.94%)	194 (14.13%)	90 (13.53%)	0.734
Classroom, Instructor-Led Training	374 (18.35%)	192 (13.98%)	182 (27.37%)	< 0.001
Live Webinars	152 (7.46%)	78 (5.68%)	74 (11.13%)	< 0.001

When asked to choose advanced topics in which they would like further education, APPs responded with a significant desire for more education in all topics listed except obstetrician-gynecologist emergencies (OB/GYN) (Table 2). Both groups demonstrated the most interest in education on procedures and documentation and the least interest in geriatrics and opiate prescribing.

**Table 2:** Self-identified preferences for additional education APP: advanced practice provider; OB/GYN: obstetrician/gynecologist

Topic	Total (n=2038)	Physician (n=1373)	APP (n=665)	p-value
Procedures	1136 (55.74%)	695 (50.62%)	441 (66.32%)	< 0.001
Documentation Tips	1002 (49.17%)	611 (44.50%)	391 (58.80%)	< 0.001
Toxicology	815 (39.99%)	513 (37.36%)	302 (45.41%)	< 0.001
Pediatrics	791 (38.81%)	496 (36.13%)	295 (44.36%)	< 0.001
Neurological Conditions	780 (38.27%)	479 (34.89%)	301 (45.26%)	< 0.001
Trauma	751 (36.85%)	440 (32.05%)	311 (46.77%)	< 0.001
OB/GYN Emergencies	664 (32.58%)	426 (31.03%)	238 (35.79%)	0.034
Infectious Disease Conditions	692 (33.95%)	399 (29.06%)	293 (44.06%)	< 0.001
Cardiovascular Conditions	669 (32.83%)	389 (28.33%)	280 (42.11%)	< 0.001
Respiratory Conditions	519 (25.47%)	255 (18.57%)	264 (39.70%)	< 0.001
Opiate Prescribing and Education	495 (24.29%)	267 (19.45%)	228 (34.29%)	< 0.001
Geriatrics	344 (16.88%)	199 (14.49%)	145 (21.8%)	< 0.001

## Discussion

This is the largest study to date describing CME delivery preferences in a cohort of primarily nonacademic emergency medicine providers working for a large practice group. We found that practicing emergency medicine providers prefer to receive their CME by video and podcast much more than traditional teaching modalities (eg, classroom teaching and written materials). While these findings are probably not surprising given the recent work detailing similar trends among resident physicians [7] and medical students [8], the influence of videos and podcasts in the CME domain bears exploration. Over a decade ago, the CME literature spoke of the potential of online technology to efficiently deliver CME, though, at that time, it had not yet displaced traditional CME [4]. This work described how practicing clinicians value the ability to quickly find information in formats that best suit their learning needs (eg, podcast, video, mobile device) and led to more recent work suggesting that the technology preferences of the millennial generation are driving a shift toward mobile technology in CME [3]. Our findings build on these studies and suggest that the predicted shift to video and audio education has already taken place within practicing emergency providers.

This shift has broad ramifications for educators and scholars interested in emergency medicine providers’ ongoing education once they leave structured education (ie, residency or fellowship training). While video and audio lead the way on CME consumption, our understanding of how these modalities work is lagging. If to optimize learning, we are to carefully analyze the nature of the content to be learned and its relation to the preferred technology characteristics [9], we must invest significantly in research that examines these delivery modalities to determine what learning environments provide appropriate knowledge translation [10]. Further, we must consider a broader approach to designing a digital CME curriculum based on our current understanding of the nature of human learning [10]. For example, including opportunities for the listener to pause to answer a question can increase knowledge retention when listening to a podcast [11]. While some CME content providers are making strides in this direction, the work is just beginning.

Additionally, our findings demonstrated that APPs preferred in-person learning compared to physicians, and they prefer ongoing procedural training. These preferences may be due to their training backgrounds (ie, medical school training versus APP training) and the lack of postgraduate training of most APPs in clinical practice [12]. Our data set did not delineate APPs into groups of nurse practitioners and physician assistants, as their training varies in terms of approach and hours.

For CME content creators, the data would support targeted in-person learning sessions with an increased focus on optimizing the delivery of online content. Based on our results, this content is most likely to be consumed if delivered in a vlog or podcast format. In-person CME can be an expensive venture in an already established market. In-person learning opportunities often come with the benefits of social interactions within the emergency medicine community, chances for collaboration, networking opportunities, and destination travels. On the other hand, live in-person CME often requires a significant amount of money and time from both the groups providing them and the providers consuming them. Further, the sense of connection to the emergency medicine community that comes with in-person CME meetings might also be experienced through podcast listening [13]. Our findings, and the shifting preferences of the emergency medicine community that they represent, suggest there may be space to blend in-person and remote learning opportunities in the future.

An easy first step might be culling in-person content for material that can be delivered in high-yield podcasting and vlog formats. Additionally, in-person sessions should be optimized to include content that cannot be handled virtually (eg, procedural practice, simulation). The careful selection and training of faculty delivering content in those settings will yield the best results for CME attendees.

Limitations

This survey-based study is not without limitations. First, the survey was collected by an emergency medicine group that offers an array of continuing education content, including remote, online, podcasts, and in-person content. Due to this, the providers responding to the survey may be biased based on the groups' offerings, and it may not represent a broader, more generalized practice of emergency medicine. This limitation, however, may be mitigated by the diversity of practice locations and environment, in addition to the number of overall respondents. Second, the survey does not detail the respondents’ demographics, limiting some of the insight from the data set in terms of gender, age, and other group preferences. Future works can use this initial data set as a foundation to investigate changes in CME delivery preferences as a general trend and specifically among targeted demographics to gain insight into how medicine can continue to deliver materials and topics in a manner the learner prefers. Third, nurses and prehospital providers were not included in this trial, limiting the generalizability to these groups. These data were obtained before the coronavirus disease 2019 (COVID-19) pandemic. Given the significant global changes and move to remote learning, how these results generalize and how providers’ preferences have already changed is unknown. Finally, our participants’ preferences should be viewed in the context of the literature on the limitations of self-assessment and judgments of learning [14]. As such, these findings should guide future studies rather than be viewed as guidance on optimizing CME delivery.

## Conclusions

This study is the first-of-its-kind evaluation of providers’ educational preferences practicing emergency medicine in the postgraduate setting. Our findings can play a vital role in the future planning of educational events regarding topics to be covered and delivery methods given learner preferences. The results from this study can also be used in further research evaluating the various demographics of those who practice emergency medicine today.

## Appendices

**Table 3:** General Survey APP: advanced practice provider; OB/GYN: obstetrics and gynecology

1. Are you a Physician or APP?
2. In which service line do you practice?
Emergency Medicine
Other
3. Please choose an advanced topic for which you would like education.
Procedures
Documentation Tips
Toxicology
Pediatrics
Neurological Conditions
Trauma
OB/GYN Emergencies
Infectious Disease Conditions
Cardiovascular Conditions
Respiratory Conditions
Opiate Prescribing and Education
Geriatrics
Other
4. What are our preferred methods of receiving educational material?
Videos
Podcasts
Presentation Slides with Audio Narration
Written Material/Articles
Infographics
Classroom, Instructor-Led Training
Live Webinars
